# Supplementary figures and images for: PPARγ attenuates cellular senescence of alveolar macrophages in asthma-COPD overlap
Source: Respir Res. 2024 Apr 20;25:174. doi: 10.1186/s12931-024-02790-6 (PMC11032609; doi:10.1186/s12931-024-02790-6)

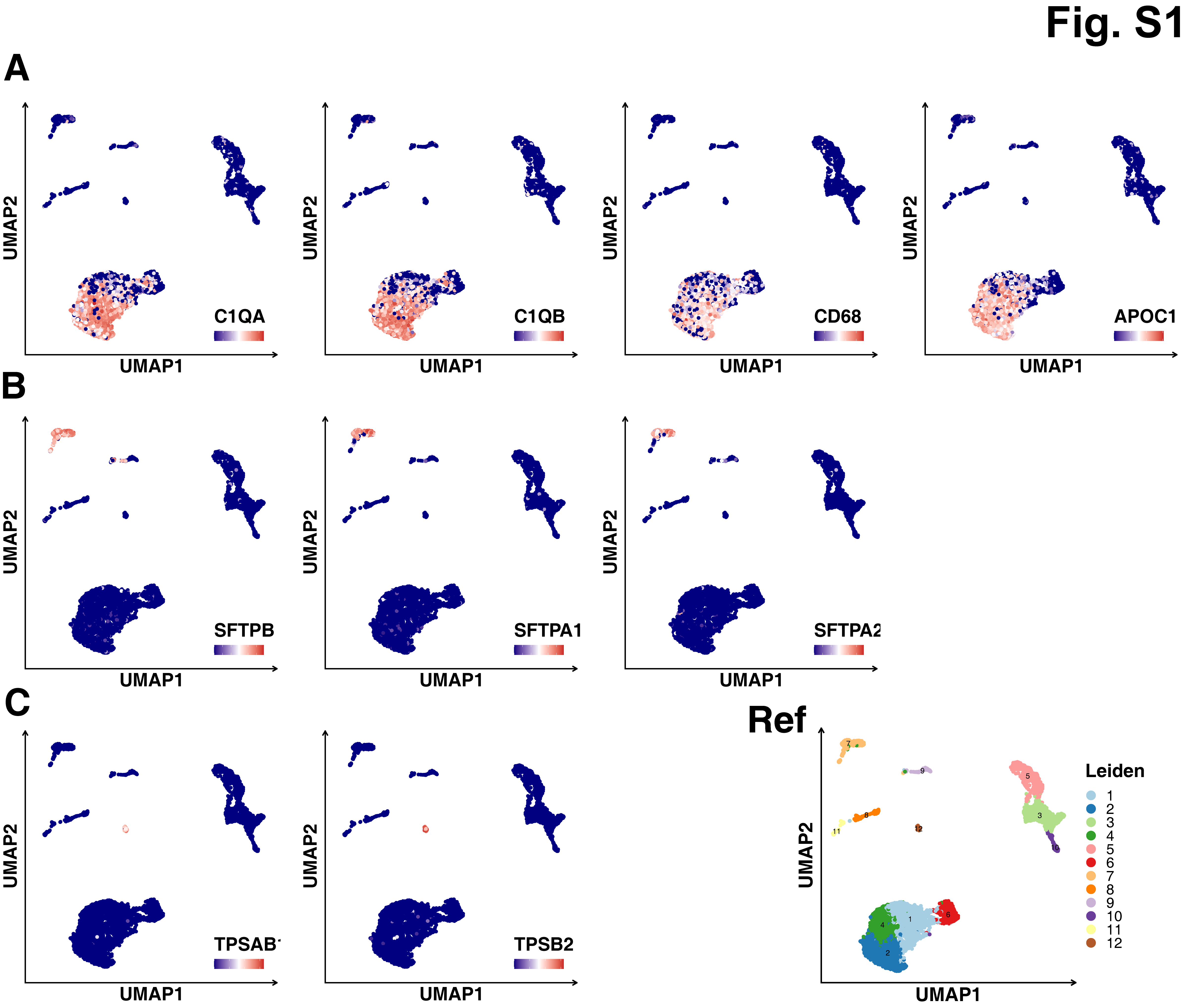

Supplement: Supplementary file 1 — Supplementary Material 1 [file 12931_2024_2790_MOESM1_ESM.tif]

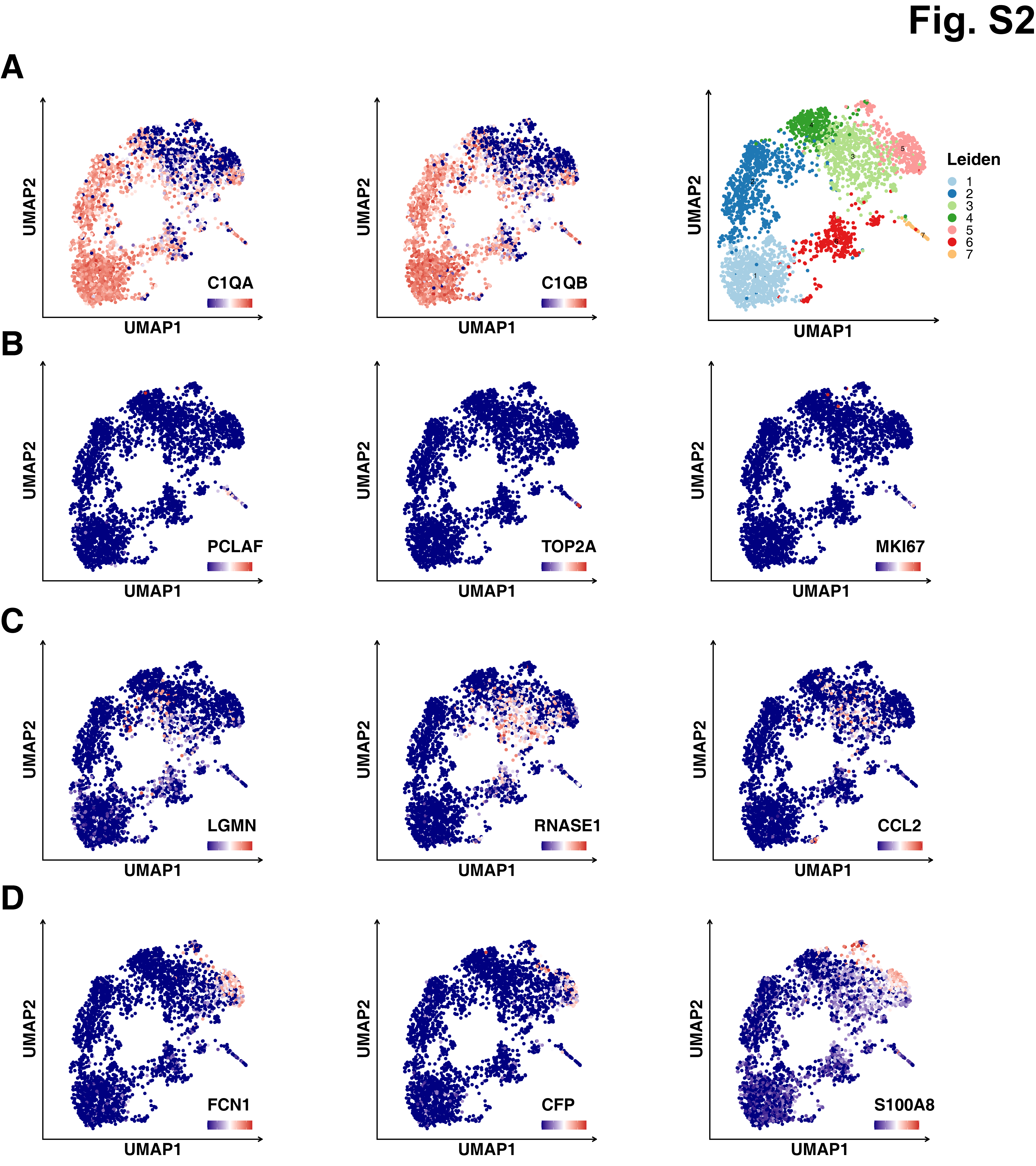

Supplement: Supplementary file 2 — Supplementary Material 2 [file 12931_2024_2790_MOESM2_ESM.tif]

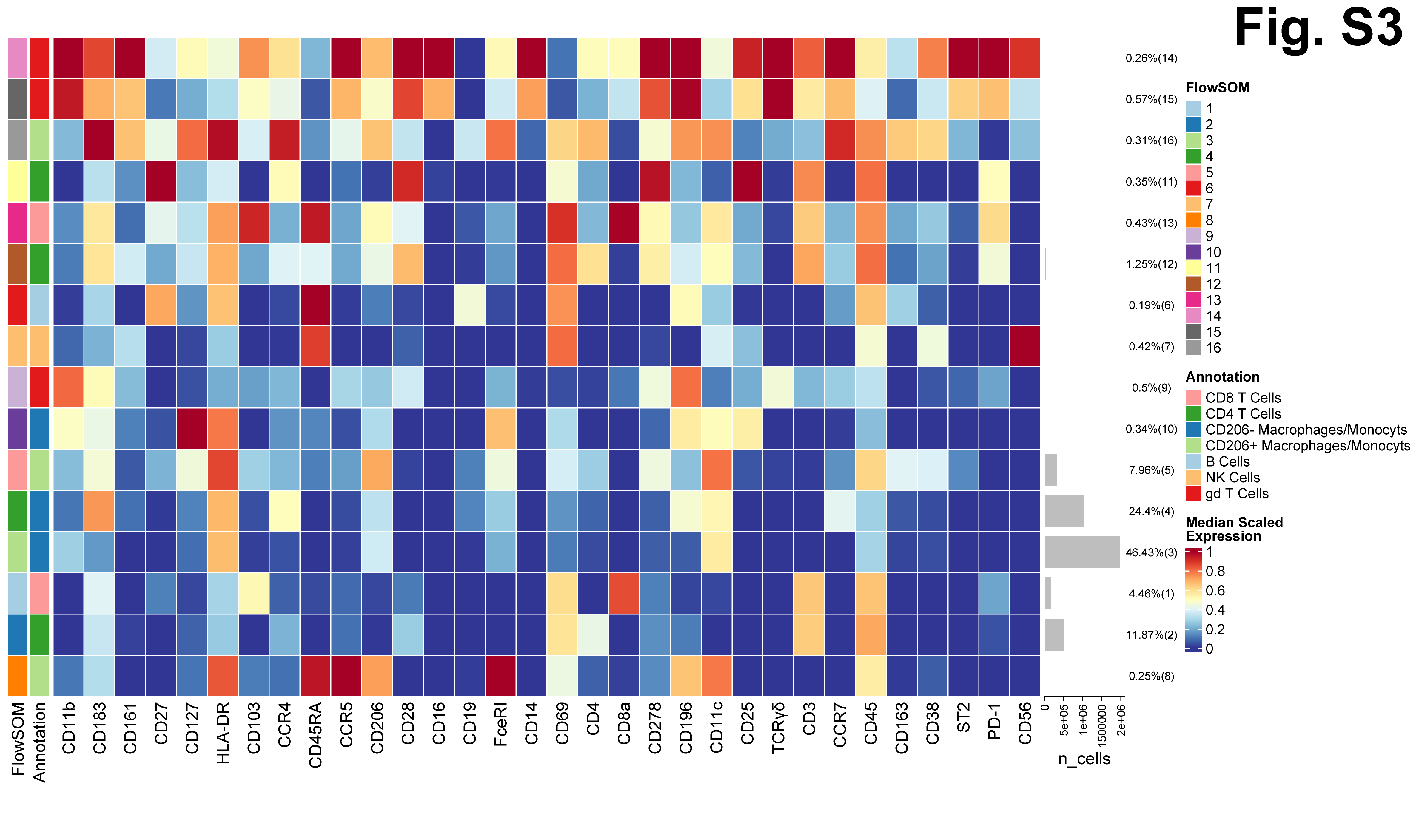

Supplement: Supplementary file 3 — Supplementary Material 3 [file 12931_2024_2790_MOESM3_ESM.tif]
